# Analyses of cytokine gene expression and fecal microbiota in a patient with Cronkhite‐Canada syndrome successfully treated with prednisolone

**DOI:** 10.1002/deo2.222

**Published:** 2023-05-08

**Authors:** Hajime Honjo, Yasuhiro Masuta, Yasuo Otsuka, Sho Masaki, Kosuke Minaga, Masatoshi Kudo, Tomohiro Watanabe

**Affiliations:** ^1^ Department of Gastroenterology and Hepatology Kindai University Faculty of Medicine Osaka Japan

**Keywords:** Cronkhite–Canada syndrome, microbiota, cytokine, prednisolone, polyposis

## Abstract

Although prednisolone treatment is effective in Cronkhite–Canada syndrome (CCS), its mechanisms of action are poorly understood. We performed analyses of cytokine expression and fecal microbiota in a patient with the concurrent occurrence of CCS and rectal cancer, in whom regression of polyposis was achieved by prednisolone. Regression of CCS polyps was accompanied by downregulation of proinflammatory cytokine expression and alterations in microbiota composition; a decrease in *Bacteroides fragilis* and *Peptostreptococcus anaerobius* with the promotion of inflammation. We could not completely exclude the possibility that alterations in fecal microbiota composition might be influenced by the presence of advanced cancer. However, this case suggests that the administration of PSL might lead to the regression of CCS polyps through alterations in gut microbiota composition and suppression of proinflammatory cytokine responses.

## INTRODUCTION

Cronkhite‐Canada syndrome (CCS) is a rare, non‐hereditary polyposis of the gastrointestinal (GI) tract.^1^ Typical CCS is characterized by skin lesions and multiple GI polyps.^1^ Inflammatory, hamartomatous, and adenomatous polyps are present in the GI tract of CCS patients.^1^ Although prednisolone (PSL) is widely used for the regression of CCS polyps, its mechanisms of action have been poorly understood.^1^ Infiltration of immune cells in CCS polyps strongly suggests pathogenic roles played by cytokines. Given that the GI tract is exposed to the intestinal microbiota, proinflammatory cytokine responses against intestinal microbiota are likely to be involved in CCS. We hypothesized that regression of CCS polyps by PSL was mediated by suppression of proinflammatory cytokine responses against intestinal microbiota. To examine this possibility, we performed cytokine gene expression and fecal microbiota analyses in a CCS patient with the concurrent occurrence of rectal cancer and successfully treated with PSL.

## CASE REPORT

**FIGURE 1:**
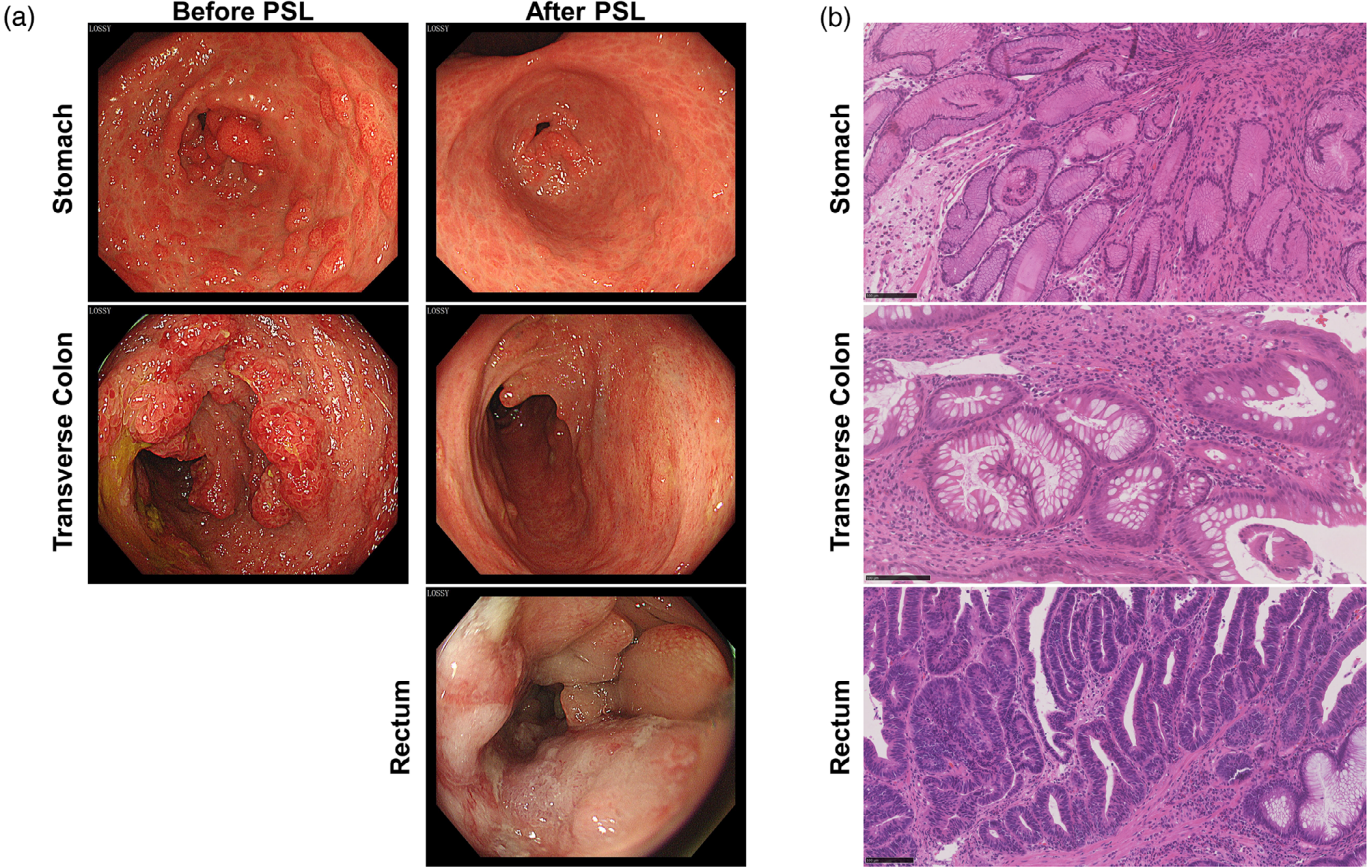
Endoscopic and pathological findings of a patient with Cronkhite‐Canada syndrome. (a) Multiple reddish polyps and edematous adjacent mucosa are seen in the gastric body and antrum before the prednisolone (PSL) treatment (Top panel). Colonoscopy reveals multiple erythematous elevated lesions in the entire colon before the PSL treatment (middle panel). Regression of Cronkhite–Canada syndrome polyps was achieved after the PSL treatment. Type III advanced cancer was detected in the rectosigmoid colon (bottom panel). (b) Pathological examination results obtained from gastric and colonic elevated lesions reveal hamartomatous polyps with cystic dilatation of glands containing intraluminal mucin and infiltration of immune cells into the submucosa. Well‐differentiated adenocarcinoma was detected in the rectal biopsy samples. Scale bar, 100 µm.

A 66‐year‐old male with alopecia, skin pigmentation, and nail atrophy was admitted. Esophagogastroduodenoscopy revealed multiple reddish polyps and an edematous adjacent mucosa in the stomach (Figure 1a). Colonoscopy revealed multiple elevated erythematous lesions throughout the colon (Figure 1a). Pathological examination of the elevated gastric and colonic lesions revealed hamartomatous polyps with cystic dilatation of glands containing intraluminal mucin and infiltration of immune cells into the submucosa (Figure 1b). These findings are consistent with those of CCS.^1^ He was treated with oral PSL (30 mg/day). Regression of CCS polyps was observed in both the stomach and colon three months after PSL treatment (Figure 1a). The disappearance of CCS polyps revealed the presence of type III advanced cancer in the rectosigmoid colon. Well‐differentiated adenocarcinoma was detected in rectal biopsy samples (Figure 1b), and he underwent lower anterior resection for rectal cancer.

**FIGURE 2:**
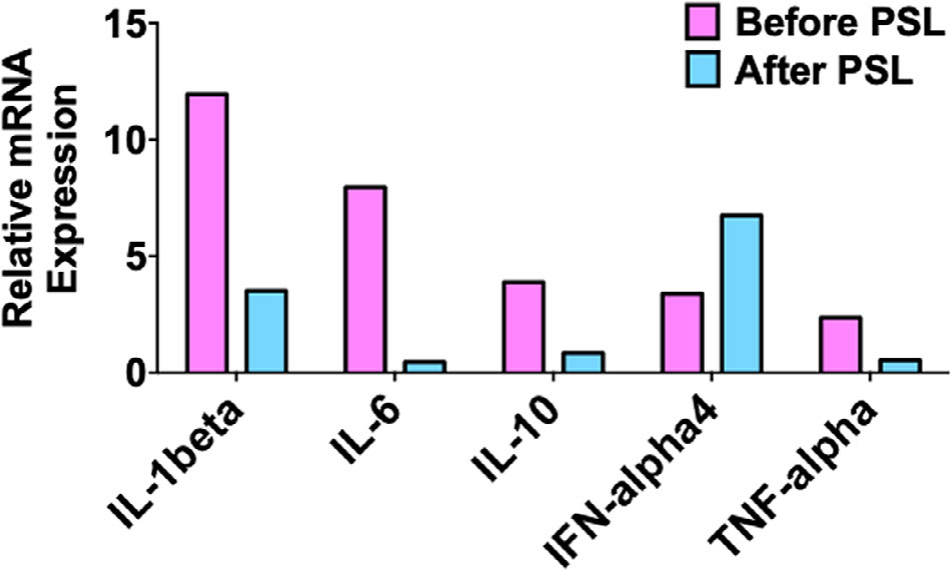
Cytokine analyses in a patient with Cronkhite‐Canada syndrome. Colonic biopsy samples obtained before and after treatment with prednisolone (PSL) were subjected to quantitative reverse transcription polymerase chain reaction analyses. Messenger RNA (mRNA) expression of interleukin (IL)‐1β, IL‐6, IL‐10, interferon (IFN)‐α4, and IL‐10 was shown.

**FIGURE 3:**
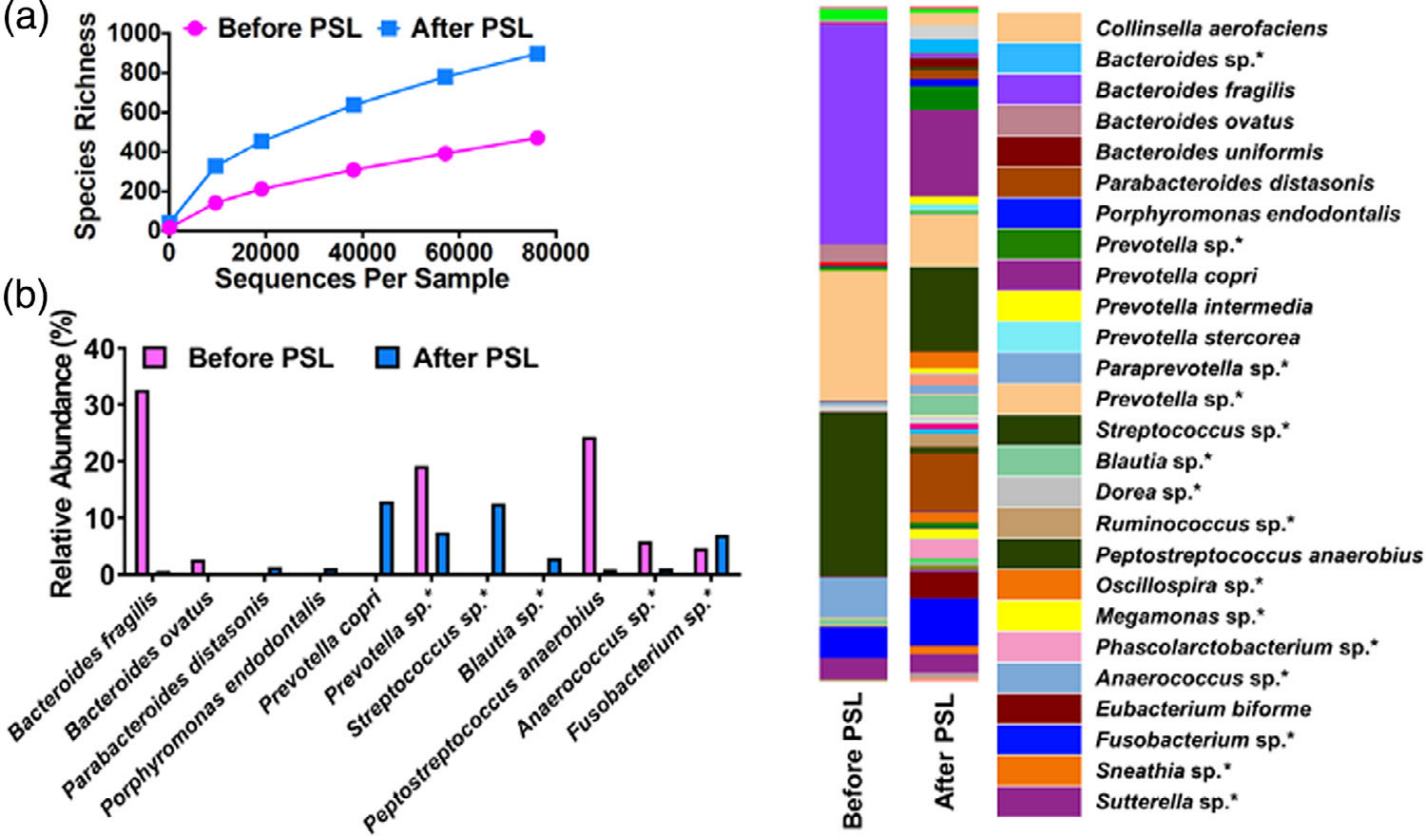
Fecal microbiota analyses in a patient with Cronkhite‐Canada syndrome. Fecal samples obtained before and after treatment with prednisolone (PSL) were subjected to 16S ribosomal RNA sequencing. (a) Rarefaction analysis of alpha diversity in fecal samples before and after PSL treatment. (b) Relative abundance of fecal microbial composition at the species level before and after PSL treatment. *Species unidentified by sequence analyses.

Colonic biopsy and fecal samples were obtained before and after PSL treatment to evaluate the involvement of proinflammatory cytokine responses and intestinal dysbiosis as described previously.^2^, ^3^ Later samples were collected prior to surgery. Ethical permission for this study was approved by the Review Boards of Kindai University Faculty of Medicine. PSL treatment markedly reduced messenger RNA expression of proinflammatory cytokines (interleukin [IL]‐1β, IL‐6, and tumor necrosis factor [TNF]‐α; Figure 2). In contrast, interferon‐α4 expression was increased after PSL treatment. Unexpectedly, PSL treatment increased the microbial diversity in rarefaction analyses of alpha diversity (Figure 3a). Remarkable alterations in the microbiota composition were observed in the samples before and after PSL treatment. A substantial reduction in the abundance of *Bacteroides fragilis* and *Peptostreptococcus anaerobius* was observed after PSL treatment (Figure 3b). In contrast, PSL treatment increased the proportion of *Prevotella copri* and species unidentified *Streptococcus*. Thus, PSL administration led to the regression of CCS polyps, which was associated with a decrease in proinflammatory cytokine expression and alteration in microbial composition.

## DISCUSSION

Although the etiology of CCS and CCS‐associated cancer is poorly defined, marked responses to PSL and TNF‐α inhibitors implicate the involvement of proinflammatory cytokine responses.^1^, ^4^ In addition, recent reports identified intestinal dysbiosis as one of the risk factors for CCS because the replacement of intestinal bacteria by fecal microbiota transplantation or gut sterilization by antibiotics is effective in CCS.^5^, ^6^ These findings led us to hypothesize that PSL or TNF‐α‐inhibitors might achieve regression of CCS polyps through suppression of proinflammatory cytokines induced by intestinal dysbiosis. In this study, we provide evidence that regression of CCS polyps by PSL was accompanied by the downregulation of proinflammatory cytokine responses and alterations in intestinal microbiota composition. It should be noted, however, that such mechanisms of action on CCS by PSL need to be examined in a large number of patients with this disorder.

Colonic messenger RNA expression of IL‐1β, IL‐6, IL‐10, and TNF‐α was markedly decreased after PSL treatment whereas expression of interferon‐α4 was increased. Thus, one mechanism of action by PSL on CCS is the downregulation of prototypical proinflammatory cytokine responses, but not the type I interferons. Moreover, immunoregulatory IL‐10 response was unlikely to be involved in the PSL‐mediated regression of CCS polyps.^7^ Interestingly, the regression of CCS polyps by PSL was accompanied by increased microbial diversity and alteration of microbial composition in this case. The dramatic alterations in the gut microbial community composition by PSL were specific to CCS since gut microbial diversity after induction of remission by PSL was comparable in patients with autoimmune pancreatitis.^3^ Therefore, we elucidated a part of molecular mechanisms of action by PSL on CCS; PSL mediates regression of polyposis not only through suppression of proinflammatory cytokines but also through alterations in intestinal microbiota composition. The link between proinflammatory cytokines triggered by dysbiosis and the pathogenesis of CCS can be further investigated by studies in a large number of patients with CCS.

Our microbiota analyses revealed decreases in two bacterial species after PSL treatment; the proportions of *B. fragilis* and *P. anaerobius* were markedly decreased. Fecal samples from patients with colorectal cancers and precancerous colon polyps have been shown to be rich in *B. fragilis* and *P. anaerobius*.^8^, ^9^ Recent studies provide evidence that colonization of these two bacteria promotes oncogenic processes through the acceleration of proinflammatory cytokine responses.^8^, ^9^ Thus, the regression of CCS polyps by PSL was accompanied by alterations in intestinal microbiota composition: a decrease in *B. fragilis* and *P. anaerobius* with the promotion of inflammation and oncogenesis. Moreover, alterations in intestinal microbiota composition by PSL are likely to change amounts of microbiota‐derived metabolites including short‐chain fatty acids with the ability to regulate cytokine responses.^10^ Therefore, it would be intriguing to examine cytokine responses and alterations in intestinal microbiota composition and in microbiota‐derived metabolites in a large number of patients with CCS.

To the best of our knowledge, no studies have provided the combined data of in situ cytokine expression and microbiota composition in CCS. However, these data were obtained in a patient bearing CCS‐associated cancer. We did not analyze intestinal microbiota compositions in the sample after the surgical resection of rectal cancer. Thus, we cannot exclude the possibility that the presence of rectal cancer might have affected alterations in fecal microbiota compositions in this case. Given the fact that all analyses were performed by utilizing samples before and after the PSL treatment in the CCS patient bearing concurrent occurrence of rectal cancer, we have to be cautious regarding the interpretation of these data, especially in the context of polyposis in CCS alone. Therapeutic effects by PSL or TNF−α‐inhibitors with the ability to suppress proinflammatory cytokines are established in CCS.^4^ Furthermore, the effectiveness of the replacement of intestinal microbiota was reported in CCS.^6^ Thus, it is likely that proinflammatory cytokine responses triggered by intestinal dysbiosis are involved in the development of CCS and that PSL mediates regression of CCS polyps via suppression of such responses. Notably, intestinal dysbiosis might also accelerate the development of CCS‐associated cancer since the colonization of *B. fragilis* and *P. anaerobius* with the promotion of inflammation and oncogenesis is enriched in this case before PSL treatment. Thus, our analyses of cytokine and microbiota support the involvement of the inflammatory polyp‐adenoma‐carcinoma sequence in CCS‐associated cancer.^1^

## CONFLICT OF INTEREST

None.
